# Main and Minor Types of Collagens in the Articular Cartilage: The Role of Collagens in Repair Tissue Evaluation in Chondral Defects

**DOI:** 10.3390/ijms222413329

**Published:** 2021-12-11

**Authors:** Lourdes Alcaide-Ruggiero, Verónica Molina-Hernández, María M. Granados, Juan M. Domínguez

**Affiliations:** 1 Departamento de Medicina y Cirugía Animal, Facultad de Veterinaria, Universidad de Córdoba, Hospital Clínico Veterinario, Campus de Rabanales, Ctra. Madrid-Cádiz Km 396, 14014 Córdoba, Spain; jmdominguez@uco.es; 2 Fundación García-Cugat, Plaza Alfonso Comín 5-7, 08023 Barcelona, Spain; 3 Departamento de Anatomía y Anatomía Patológica Comparadas y Toxicología, Facultad de Veterinaria, Universidad de Córdoba, Edificio de Sanidad Animal, Campus de Rabanales, Ctra. Madrid-Cádiz Km 396, 14014 Córdoba, Spain; b62mohev@uco.es

**Keywords:** hyaline articular cartilage, main collagens, minor collagens, chondral defect repair

## Abstract

Several collagen subtypes have been identified in hyaline articular cartilage. The main and most abundant collagens are type II, IX and XI collagens. The minor and less abundant collagens are type III, IV, V, VI, X, XII, XIV, XVI, XXII, and XXVII collagens. All these collagens have been found to play a key role in healthy cartilage, regardless of whether they are more or less abundant. Additionally, an exhaustive evaluation of collagen fibrils in a repaired cartilage tissue after a chondral lesion is necessary to determine the quality of the repaired tissue and even whether or not this repaired tissue is considered hyaline cartilage. Therefore, this review aims to describe in depth all the collagen types found in the normal articular cartilage structure, and based on this, establish the parameters that allow one to consider a repaired cartilage tissue as a hyaline cartilage.

## 1. Introduction

Collagens are the most abundant proteins in mammals accounting for approximately 30% of total protein mass [1]. More than two-thirds of the dry weight of adult articular cartilage, over three-fourths of the dry weight of human skin, over 90% of human tendon and corneal tissues, and almost 80% of the organic matter in bones are composed of collagen, a major component of connective tissues [2,3]. Collagen is approximately 60% of the dry weight of hyaline cartilage, being the main protein in the cartilage extracellular matrix (ECM) composition [4,5]. Since the discovery of the first collagen [6], more than 26 new collagens have been revealed [1,7,8,9,10].

In articular cartilage, characterized by having a hyaline structure, numerous subtypes of collagens have been identified. The main, most abundant collagens, which are mostly studied by researchers are type II, IX, and XI collagens [11,12]. Type III, IV, V, VI, and X collagens are less abundant in cartilage, which is why some authors highlight them and others do not [11,12,13]. Even so, the presence of these minor collagens in healthy articular cartilage is reliable. However, the quantity of some of these minor collagens in healthy cartilage is still controversial. In addition, some researchers mentioned that type XII, XIV, XVI, XXII, and XXVII collagens are also part of articular cartilage [13], although the information given about them is very brief. As well as all the collagens mentioned above, type I collagen can sometimes be found in articular cartilage. However, this should not occur in healthy articular cartilage as it indicates the presence of fibrotic connective tissue [14].

When a chondral defect is treated, an attempt is made to repair the tissue by making the characteristics of the new cartilage as similar as possible to normal hyaline cartilage. To assess the effectiveness of the treatment that has been used, it is necessary to assess the quality of the repaired tissue, and for this purpose we need to establish a threshold that allows us to identify the repaired cartilage as hyaline cartilage. Moreover, it is essential to conduct a study of the collagens present in the repaired cartilage tissue to conduct a good assessment of the quality of the chondral repair.

Therefore, the purpose of this review is to describe in detail all of the collagens that can be found in articular cartilage, as well as describe collagen fibrils assessment considerations in order to identify repaired hyaline cartilage in chondral defects.

## 2. Articular Cartilage

Hyaline articular cartilage is a highly specialized connective tissue, whose main function is to provide a smooth and lubricated surface for the joint and to facilitate the transmission of loads with a low coefficient of friction [15,16,17]. Articular cartilage is composed of hyaline tissue including a dense ECM with a low distribution of chondrocytes [18]. Unlike most tissues, it lacks blood vessels, lymphatics and nerves, and in addition, its cells have a low replication potential, so the cartilage repair capacity is limited [19,20,21].

ECM consists of interlocking mesh of water, collagens, and proteoglycans (PG), as well as smaller amounts of non-collagen proteins. All components combined generate a single viscous material, optimized to support loads [15,22]. Chondrocytes are the only highly specialized cells found in cartilage that constitute between 1–5% of cartilage volume. These cells play a pivotal role providing mechanical support [11,23].

### 2.1. Cartilage Zones

**Figure 1:**
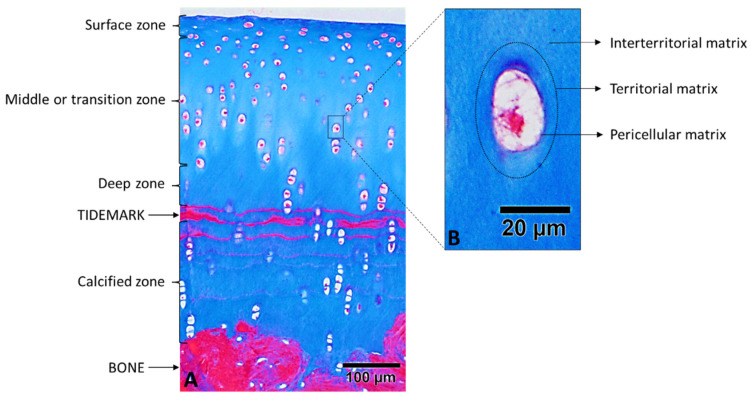
Masson’s trichrome staining of a representative section of healthy hyaline cartilage. (**A**) Cartilage zones. (**B**) Regions of the ECM.

Adult cartilage has an architecture divided into zones (Figure 1A), which vary depending on the biochemical composition of ECM, cell density and morphology, and cellular and ECM metabolism [24].

The surface zone is the outermost and thinnest of all layers and constitutes approximately 10% to 20% of the thickness of the joint cartilage. It is composed of a relatively high number of oval shaped chondrocytes parallel to the surface of the joint. Chondrocytes in this area synthesize a high concentration of collagen and a low concentration of proteoglycans. This zone has the highest water content. The collagen fibers in this zone are heavily packaged and are also aligned parallel to the joint surface, to provide the greatest resistance to traction and shear. The superficial zone is in contact with synovial fluid and is responsible for most of the traction properties of cartilage [11,15,19,25,26].

Immediately underneath the surface zone is located the middle or transition zone, which provides an anatomical and functional bridge between the superficial and deep zones. The middle zone represents 40% to 60% of the total volume of cartilage and contains thicker PG and collagen fibrils. In this zone, collagen is organized obliquely, and chondrocytes are spherical and have low density. Fundamentally, the middle zone is the first line of resistance to compression forces [11,15,19,25,26].

Then, the deep zone involves the connection between the cartilage tissue and subchondral bone. The deep zone is responsible for providing the greatest resistance to compression forces since the collagen fibrils are arranged perpendicular to the joint surface. This zone contains the largest diameter collagen fibrils in radial arrangement, the highest content of PG and the lowest concentration of water. Chondrocytes are larger and are usually available in a columnar orientation, parallel to the collagen fibers and perpendicular to the surface. The deep zone represents approximately 30% of the joint cartilage volume. Between the deep zone and the calcified cartilage, we find the tidemark that distinguishes both areas [11,15,19,25,26].

The calcified cartilage zone plays an integral role in the fixation of cartilage to the bone by anchoring collagen fibrils from the deep zone to the subchondral bone. In this zone, the cell population is scarce, and chondrocytes are hypertrophic, which makes metabolic activity very low [11,15,19,25,26].

### 2.2. Regions of the Extracellular Cartilage Matrix

In addition to the zoned architecture, the matrix consists of several regions. The ECM can be divided into three regions, depending on the proximity to chondrocytes, the composition, the diameter, and organization of collagen fibrils: pericellular, territorial and interterritorial (Figure 1B) [15]. The pericellular matrix (PCM) is a thin layer adjacent to the cell membrane that completely surrounds the chondrocyte, playing an important role in the transduction of signals and other aspects of cartilage [24]. The territorial matrix surrounds the PCM, this being thicker and protecting the cartilage cells against mechanical stress. In addition, it contributes to the elasticity of the cartilage structure and its ability to withstand loads. The interterritorial matrix is the biggest, contributing more to biomechanical properties of articular cartilage [11,15,23].

## 3. Collagens

Collagen is the major ECM molecule that self assembles into cross striated fibrils, provides support for cell growth and is responsible for the mechanical resilience of connective tissues. To date, between 28 and 29 types of collagens have been described [27,28,29].

Collagen belongs to the family of glycoproteins that are characterized by signature features. Each polypeptide chain has a repeating sequence of amino acids [Gly-X-Y]n, with and without interruptions (those with interruptions contain numerous residues). Furthermore, the X and Y positions are frequently occupied by proline and its hydroxylated form, 4-hydroxyproline, respectively. Finally, the right-handed tripe helix is formed from three left-handed polyproline α-chains of identical length, which gives collagen a unique quaternary structure [8,9,27,30].

### 3.1. Biosynthesis of Collagens Fibers

**Figure 2:**
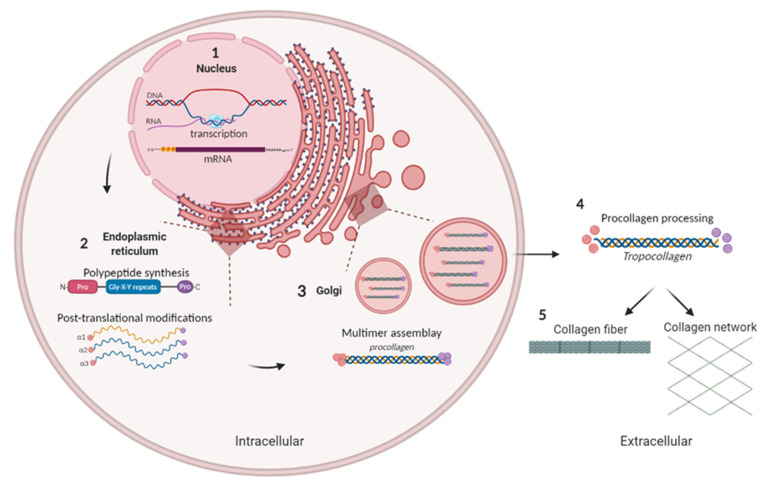
Schematic representation of collagen biosynthesis. (1) Gene transcription. (2) Formation of α-chains. (3) Formation of triple helix procollagen and secretion into extracellular space. (4) Procollagen processing and formation of tropocollagen. (5) Association of tropocollagen molecules to form collagen structures.

Collagen fibrils biosynthesis begins with the genetic transcription of genes (Figure 2(1)) within the nucleus to the aggregation of collagen heterotrimers into large fibrils [31].

Inside the rough endoplasmic reticulum (RER), the assembly of mainly three amino acids (glycine, proline or its derivative hydroxyproline, and lysine) gives rise to the formation of polypeptide chains (α chains) (Figure 2(2)). In the Golgi, three of these α chains are assembled one around the other, along a central axis, to generate a procollagen molecule in the form of right-handed triple-helix (Figure 2(3)) [32,33].

Subsequently, procollagen is secreted into the extracellular space, giving rise to tropocollagens units (Figure 2(4)). Once in the extracellular space, molecular processing is different depending on the type of collagen in question and the supramolecular structure that it must form in a tissue. In general, in the extracellular space, several tropocollagen molecules associate to form fibrils and fibers (Figure 2(5)) [31,32,34].

### 3.2. Classification of Collagen Types

Collagens can be grouped based on their structure, function, and tissue distribution. They are designated by Roman numerals according to the order of their discovery and Greek letters to identify the chains, bands, and higher molecular weight components. There are homotrimers, formed by three identical chains, or heterotrimers, formed by two/three different chains [27,32,35].

**Figure 3:**
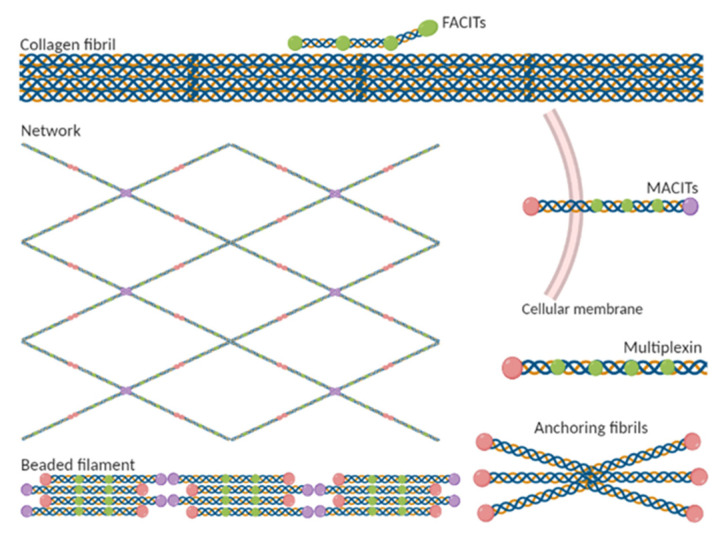
Classification of collagen types based on their structural and organisation.

The different types of collagens and their structure are crucial to provide mechanical stability, elasticity, and strength to tissues and organs. Following a classification based on collagen function and composition, several groups are distinguished (Figure 3): (1) Fibril-forming collagens; (2) Fibril-associated collagens with interrupted tripled helices; (3) Collagens forming networks; (4) Transmembrane collagens; (5) Multiplexins; (6) Anchor fibers; and (7) Beaded filament-forming collagens [9,32].

(1) The classical fibril-forming collagens include types I, II, III, V and XI collagens. They are characterized by appearing as periodic fibrils with an indeterminate in length, depending on the tissue and developmental stage, and range in diameter from 12 nm to 500 nm [36]. All fibril-forming collagens are composed of a large continuous triple helix bordered by the N- and C-propeptide referred as the NC1 domain. The N-propeptide is divided into sub-domains: a short sequence (NC2) that links the major triplex helix to the minor one, and a globular N-terminal end (NC3) [8]. This collagen is the most abundant collagen in vertebrates, and it plays a structural role by contributing to the molecular architecture, shape and mechanical properties of tissues [37].

(2) The types IX, XII, XIV, XVI, XIX and XX collagens belong to the fibril-associated collagens with interrupted tripled helices (FACITs). They are relatively short collagens, with interruptions in the triple helical domain and can be found at the surface of collagen fibrils. These molecules are mostly heterotrimers and carry a glycosaminoglycan side chain [8,31]. These collagens are involved in the integrity and stability of the ECM, modulating the formation and size of the collagen fibrils and controlling cellular organization in the ECM [38].

(3) Collagens forming networks are longer than classical fibril-collagens and can give rise to different kinds of networks depending on the collagen type [10]. These collagens include collagen types IV, VI, VIII and X. They are non-fibrillar collagens that aggregate linearly or laterally to form open networks. The collagen networks act as supporting structures for cells and tissues, serve as selective molecular filters and barriers and function as anchor for neighboring cells [39].

(4) The group of transmembrane collagens (MACITs) is comprised of type XIII, XVII, XXIII and XV collagens. These collagens are homotrimers of an α-chain which contains an N-terminal intracellular domain, a hydrophobic transmembrane stretch, and a large extracellular C-terminus. All members of this group are also shed from the cell surface, generating soluble forms. They are found in numerous cell types and stand out for its cell adhesive properties [10,40].

(5) Type XV and XVIII collagens are multiplexins that are non-fibrillar collagens and have multiple interruptions within its collagenous domain enabling more structural flexibility [41]. These collagens occur in the epithelial and endothelial basement membrane zones of a wide variety of tissues. Their biological roles are essentially separate, that of collagen XV in the muscle and that of collagen XVIII in the eye [8].

(6) Type VII collagen is the main constituent of the anchoring fibrils, structures that mediate the adhesion of the epidermis onto the dermis. It consists of a central collagenous triple-helical domain flanked by NC1 and NC2 domains [42,43].

(7) Type VI collagen is the archetypal beaded filament-forming collagen. It is widely expressed and holds up tissue integrity. Collagen VI monomers are made up of short triple helical domains, which aggregate linearly to form beaded filaments or laterally through their globular domains, thus creating 3D networks [44,45].

## 4. Types of Collagens in Articular Cartilage

There are numerous subtypes of collagens in the articular cartilage (Table 1). In healthy joint hyaline cartilage, there are main collagens (type II, IX, and XI collagen) and minor collagens (type III, IV, V, VI, X, XII, XIV, XVI, XXII, and XXVII collagens). In articular cartilage with any damage or pathology type I collagen could be found.

### 4.1. Main Collagens of Healthy Articular Cartilage

#### 4.1.1. Type II Collagen

Type II collagen is a homotrimeric molecule of three α1(II) chains, encoded by the *COL2A1* gene, and mainly synthesized by chondrocytes and nucleus pulposus cells [46,47]. It is expressed, synthesized, and secreted into the ECM as two isoforms (IIA and IIB). These isoforms are generated in a developmentally regulated manner by alternative splicing of exon 2. Chondroprogenitor cells synthesize predominantly IIA isoforms (containing exon 2), while differentiated chondrocytes produce mainly IIB transcripts (devoid of exon 2) [46,48].

Type II collagen is a fibrillar collagen that is restricted to cartilages, vitreous and intervertebral discs [49]. The mature articular cartilage comprises more than 90–95% of cartilage collagen. Type II and type XI collagen co-polymerize with type IX collagen to form a heteropolymeric fibrillar framework that gives cartilage its tensile strength [3,50,51].

Type II collagen, together with other proteins and PG, can form complex extracellular scaffolds to bear mechanical forces, maintain physiological homeostasis, and provide anchoring sites for chondrocytes, ECM molecules, and growth factors. Degradation and reduction of type II collagen are frequently observed in osteoarthritic cartilage. The type II collagen decrease in osteoarthritic cartilage is thought to be caused by chondrocyte hypertrophy. In addition to its structural function, type II collagen is an important extracellular signaling molecule that can regulate chondrocytes proliferation, metabolism, and differentiation, similar to soluble signals [52].

#### 4.1.2. Type IX Collagen

Type IX collagen is a heterotrimer composed of α1(IX), α2(IX) and α3(IX) chains encoded by genes *COL9A1*, *COL9A2* and *COL9A3*, respectively, and belong to the group of FACITs collagens [53,54].

This collagen is found mostly in cartilages, but it also occurs in the eyes, ear, and intervertebral discs, always in co-existence with type II collagen [55].

In regard to articular cartilage, type IX collagen constitutes 1% to 5% of total collagen in adult humans and 10% of that in fetus. It is proposed to stabilize the fibrillar and proteoglycan networks via lateral association with type II and type XI collagen [3,13,56].

Several studies showed that type IX collagen may play important roles in the pathogenesis of arthritis diseases, the formation of a stable collagen network and in the maintenance of cartilage organization and integrity [57,58]. Loss of type IX collagen in aging articular cartilage may results in a weaker matrix that is more susceptible to degradation [59]. In addition, type IX collagen mutations in human have been linked with the autosomal dominant diseases, multiple epiphyseal dysplasia, characterized by short stature and severe joint pain caused by early onset of osteoarthritis (OA) [59]. Therefore, type IX collagen is crucial for the maintenance of cartilage matrix and formation of collagen meshwork [3,13,53]. The reduced level of type IX collagen may contribute to the pathogenesis of osteoarthritis [13].

#### 4.1.3. Type XI Collagen

Type XI collagen is composed of three α-chains, α1(XI), α2(XI), and α3(XI), which are encoded by *COL11A1*, *COL11A2*, and *COL2A1*, respectively [60]. The α3(XI) chain of type XI procollagen and the α1(II) chain of type II procollagen are encoded by the same gene, being α3(XI) chain, an over-hydroxylated version of α1(II) [46,61].

This collagen is a cartilage-specific ECM protein important for cartilage collagen fibril formation and for ECM organization, but it is also broadly distributed in tendons, trabecular bone, testis, trachea, skeletal muscle, placenta, ovarian, lung and the neoepithelium of the brain [60,62,63].

In fetal cartilage, type XI collagen represents around 10% of total collagen, while in adult human cartilage its presence decreases to 3% [12]. In fetal cartilage, type XI collagen consists of molecules containing three genetically distinct chains α1(XI), α2(XI), and α3(XI) in a 1:1:1 ratio. However, from mature articular cartilage, purified type XI collagen also includes about equal amounts of α1(V) and α1(XI) chains, suggesting the existence of type V/XI hybrid molecules in the matrix [64].

In cartilage, type XI collagen is a minor component of collagen fibrils, but it is essential for the interaction between PG aggregates and collagens [60]. It is polymerized to form the core of type II collagen fibrillogenesis and regulates type II fibril diameters in the cartilage [46]. Type XI collagen molecules are found in thin cartilage fibrils composed of four micro-fibrils, two of which are type II collagen and two of which are type XI collagen, surrounded by ten type II micro-fibrils [65].

Type XI collagen is the first cartilage collagen deposited by mesenchymal stem cells undergoing chondrogenic differentiation [66], suggesting its involvement in the regulation of cartilage formation [67]. It is preferentially retained at the chondrocyte surface and involved in the organization of the PCM via interaction with cartilage PG [13].

Mutations in type XI collagen cause various types of chondrodysplasias which are known as “type XI collagenopathies.” Some of these chondrodysplasias in human include Stickler syndrome type II, Marshall syndrome, and oto-spondylo-megaepiphyseal dysplasia [13,64,67]. Furthermore, it has been shown that a type XI collagen mutation results in increased degradation of type II collagen in articular cartilage [13].

### 4.2. Minor Collagens of Healthy Articular Cartilage

#### 4.2.1. Type III Collagen

Type III collagen is a homotrimer of three α1(III) chains, which are encoded by *COL3A1* gene [49].

This collagen is the second most abundant collagen type in human body, classified as one of the major fibrillar collagens. It constitutes about 5–20% of the entire collagen content in the human body [68,69]. Type III collagen is found as a major structural component in hollow organs such as large bloods vessels, uterus and bowel, tissues that must withstand stretching. It is also found as a copolymer with type I collagen in many tissues, including skin, tendon, ligament, vascular walls, periodontal ligament, and synovial membranes [70].

Type III collagen provides tensile strength and integrity for many organs, but also other different functions has been reported for this collagen. One of the earliest studies on type III collagen, carried out by Balleisen et al. [71], showed that it influences the aggregation of human platelets. Subsequent studies showed that the platelets interact with type III collagen through specific glycoproteins [72,73]. Type III collagen also functions in cell adhesion, migration, proliferation, and differentiation through its interaction with integrins [70].

Regarding the role that type III collagen plays in cartilage, there are some controversies, because we can find it both in healthy cartilage and in aged or osteoarthritic cartilage. Thus, the content of type III collagen could vary markedly at different stages of development and disease. Some studies confirmed that a small but significant amount of type III collagen becomes deposited in articular cartilage of mature joints, concentrated in the matrix surrounding chondrocytes throughout the depth of the tissue and particularly prominent in human osteoarthritic joints [74,75]. According to Hosseininia et al. [76], type III collagen in the human articular cartilage increases in the territorial matrix of aging individuals, although it remains unclear whether this increase represents a protective response to cartilage degeneration or a contributor to the pathological process. Moreover, in human OA, type III collagen is significantly up-regulated in cartilage [76]. Another study revealed that type III collagen is found in the adult human cartilage, and it has been suggested that its role is to act as a modifier of the fibril network composed of type II collagen together with other minor collagens during tissue healing [49,70]. Wang et al. showed that type III collagen is a crucial matrix constituent for the establishment of normal cartilage ECM [68].

Mutations in the *COL3A1* gene cause the vascular type of Ehlers-Danlos syndrome, which is a rare, life-threatening genetic disease. Other disease phenotypes associated with *COL3A1* include a brain abnormality characterized by frontoparietal polymicrogyria, and many fibrotic diseases [70].

#### 4.2.2. Type IV Collagen

Type IV collagen has a triple-helical structure composed of 3 of 6 different α- chains (α1, α2, α3, α4, α5, and α6) encoded by *COL4A1, COL4A2, COL4A3, COL4A4, COL4A5,* and *COL4A6*. These helical polypeptide α-chains form triple-helical isoforms, which only 3 have been identified: α1(IV)α1(IV)α2(IV) or (α112), α3(IV)α4(IV)α5(IV) or (α345), and α5(IV)α5(IV)α6(IV) or (α556). They are assembled into three major networks (α112:α112, α112:α556, α345:α345), interconnected by NC1 domain [29,77,78,79].

This collagen is identified primarily in the skin, is the most important structural component of basement membranes [31,80]. α1 and α2 chains are expressed ubiquitously in basement membranes, although type IV collagen α3, α4, α5, and α6 chains have a tissue specific distribution [80]. The α3α4α5 network has mainly been identified in lung alveoli, kidney, testis, cochlea, and eye, whereas the α5α5α6 network has been located in skin, smooth muscle cells, oesophagus, and Bowman’s capsule of the kidney [29,81].

Regarding cartilage, type IV collagen is observed in the PCM of healthy cartilage tissues but generally absent in degenerated and fibrotic cartilage tissues [79], although there is evidence that it can be found in PCM of degenerative hyaline cartilage [82]. It was observed that type IV collagen was in both in vitro engineered cartilaginous constructs and in vivo cartilage repair samples, in addition they shown that chondrocytes were capable of synthesizing type IV collagen [82].

The specific isoform of type IV collagen α112(IV) was identified as the unique type in articular cartilage. This isoform contains arrestenand canstatin that are anti-angiogenic protein fragments. Since articular cartilage is an avascular structure, type IV collagen isoform α112(IV) with their unique anti-angiogenic properties might be involved in the temporal control of vascularization during cartilage repair and in cartilage homeostasis [79].

Mutations in type IV collagen α3 to α6 chains causes Alport’s syndrome associated with glomerulonephritis, sensorineural deafness and eye abnormalities [80,81].

#### 4.2.3. Type V Collagen

The collagen type V triple helix is formed as a heterotrimer by one α1(V) chain and two α2(V) chain, which are encoded by *COL5A1* and *COL5A2* genes. It is widely distributed in tissues as α1(V)2α2(V) that integrate into fibrils of the abundant type I collagen and regulate the geometry of resulting col(I)/col(V) heterotypic fibrils [83,84]. There is a third col(V) chain, α3(V), which can be found in α1(V)α2(V)α3(V) heterotrimers and has more limited tissue distribution than α1(V)2α2(V) heterotrimers. Tissues in which the α3(V) chain have been detected in white adipose tissue, skeletal muscle, and pancreatic islets, where these chains are important for proper functioning of the adipocytes, myofibers, and pancreatic β cells, respectively [84,85].

This collagen is classified as a minor fibrillar collagen that under normal physiologic conditions assembles into heterotypic fibrils with the major fibrillar collagen type I [86], although it can also be found assembled as type V/XI collagen [87].

Despite being a minor collagen, type V collagen is an abundant protein in skin, placenta and lung, and essential for tissue elasticity and compliance [88]. Type V collagen plays a basic function in the formation of fibrillar collagen mesh and has an important role in fibrogenesis control or fiber size regulation. Furthermore, the type V collagens contribute to the linking between stromal collagen and basement membrane, being important for cellular adhesion and matrix-repairing process [89].

As regards to articular cartilage, D.R. Eyre and J.J. Wu. [50], isolated mature articular cartilage, where type XI collagens included a significate pool of α1(V) chains which implied the presence of V/XI hybrid molecules. Later, a study carried out by J.J. Wu et al. [87], proved the presence of V/XI hybrid molecules in articular cartilage showing an accumulation of collagen α1(V) chains as articular cartilage matures. Although it has been shown that the type V collagen is present in articular cartilage [90], no studies have been found that discuss its role in articular cartilage.

One of the main diseases associated with a defect in type V collagen is classic Ehlers-Danlos syndrome. It is a rare autosomal dominant connective tissue disorder that is primarily characterized by skin hyperextensibility, abnormal wound healing/atrophic scars, and joint hypermobility [91].

#### 4.2.4. Type VI Collagen

Type VI collagen is a heterotrimer that has a characteristic beaded filamentous structure of tetrameric units that consists of three different α-chains, α1(VI), α2(VI), and α3(VI), which are encoded by the genes *COL6A1*, *COL6A2*, and *COL6A3*. Recently, 3 novel subunits of type VI collagen are revealed, α4(VI), α5(VI), and α6(VI) chains encoded by the *COL6A4*, *COL6A5*, and *COL6A6* genes, which are highly homologous to the α3(VI) chain [92,93,94].

This collagen is found in almost all tissue and distributed among tissues such as skin, cornea, blood vessels, heart, lungs, adipose tissue, nervous tissues, pancreas, bones, cartilage, and muscle [45,92,93].

There is a high affinity between collagen VI and numerous ECM components, as biglycan, decorin, hyaluronan, fibronectin, perlecan and heparin, as well as with the cell membrane. Thus, type VI collagen has been hypothesized to play an essential role in mediating cell-matrix interactions as well as intermolecular interactions in various tissues [95]. In addition, this collagen participates in the maintenance of tissue integrity creating cell-matrix and matrix-matrix interactions [96].

In some studies, total type VI collagen, a putative marker of mesenchymal activation, has been suggested to be an indicator of early architectural remodeling in liver fibrosis [97,98].

In skeletal muscle, type VI collagen is present in the ECM where it functions to anchor the basement membrane to underlying interstitial tissues [99]. Mutations in the *COL6A1*, *COL6A2*, and *COL6A3* genes lead to a continuous spectrum of disorders characterized by muscle weakness and connective tissue abnormalities [100]. These mutations have been identified as causative in Ullrich congenital muscular dystrophy, Bethlem myopathy and myosclerosis myopathy [45,101].

In articular cartilage, type VI collagen is present in small amounts (1–2%) forming a network that anchors the chondrocytes to the PCM through its interaction with a wide variety of ECM proteins, including type II collagen, type XIV collagen, cartilage matrix protein or matrilin-1, hyaluronan, decorin, and fibronectin; which implies the attachment and integrity of chondrocytes [13,95,102].

In normal adult human articular cartilage, the PCM is typically defined by the exclusive presence and localization of type VI collagen around the chondrocytes [24]. Some studies confirm that type VI collagen could contribute to PCM structural integrity and mechanical properties. Furthermore, it may serve as a filter or transducer for biochemical and/or biomechanical signals from the cartilage ECM [13,95].

Studies conducted in mice showed that those with type VI collagen deficiency exhibited accelerated development of hip OA, a delayed secondary ossification process, increased chondrocyte swelling and a loss of the stiffness of the articular cartilage PCM. These finding suggest that type VI collagen plays an essential role in transmitting mechanical and osmotic stresses from the ECM to the chondrocytes [95,103].

#### 4.2.5. Type X Collagen

Type X collagen is a homotrimeric collagen which consists of three identical α1(X) chains, encoded by the *COL10A1* [104,105].

This collagen is a specific collagen in cartilage. It constitutes about 1% of total collagen in adult human articular cartilage [13]. It is synthesized by hypertrophic chondrocytes during enchondral bone formation and is found exclusively in the hypertrophic cartilage and the calcified zone of articular cartilage [106]. Thus, the 45% of the total collagens produced by mature hypertrophic chondrocytes are type X [104]. As the most widely used marker for chondrocyte hypertrophy, type X collagen in normally expressed in human OA cartilage especially in the vicinity of lesions, but not in human healthy articular cartilage [107,108]. Hypertrophic chondrocytes express a variety of proteins and enzymes as type X collagen, matrix metalloproteinase 13 (MMP13), alkaline phosphatase, which do not seem to exit in normal proliferating chondrocytes [109]. These chondrocytes increase their volume and secrete a specialized ECM rich in type X collagen. This matrix attracts blood vessels and bone precursor cells leading to bone development, but also the process of endochondral ossification has been reported in cartilaginous tumors [110].

The biological function of type X collagen in thought to maintain tissue stiffness, regulate chondrocytes metabolism and interact with hypertrophic chondrocytes [106,111]. It also facilitates the process of calcification, the normal distribution of matrix vesicles and PG within the growth plate [13,111].

Considering that ECM surrounding chondrocytes mineralizes to be replaced by bone marrow and bone, it was suggested that collagen X may be associated with the mineralization process [39]. Its expression at sites of chondrocyte hypertrophy and calcification suggest that type X collagen support endochondral bone growth and development during the degradation of ECM in cartilage [13,109].

Mutations of the *COL10A1* gene are causative for the disease Schmid type metaphyseal chondrodysplasia (MCDS; MIM 156500) impeding endochondral ossification in the metaphyseal growth plate. This leads to growth deficiency and skeletal deformities with short limbs [31]. These mutations have been also considered key in spondylometaphyseal dysplasia (SMD) which is a group of genetic skeletal disorders that show abnormal development of spine and metaphysis of long tubular bones [112].

#### 4.2.6. Type XII, XIV, XVI, XXII, and XXVII Collagens

Type XII belong to the group of FACITs collagens. Immunohistochemistry, staining and fibrillogenesis studies showed that type XII collagen can be incorporated into type I collagen fibrils in dense connective tissues and bone [113]. Type XII collagen is associated with articular cartilage and growth plate region during rat forelimb development and may be necessary for microenvironment that support hyaline cartilage formation [13,113]. Type XII collagen was also described in the secretome of human passaged chondrocytes [114]. Type XIV collagen is a large, non-fibrillar ECM protein which also belong to the group of FACITs. Immunofluorescence localization showed that type XIV collagen was prominent at the ligament-bone junction, and in bovine cartilage. Type XIV collagen localizes relatively uniformly throughout the articular cartilage but is absent from growth plate regions [13]. Both, type XII and XIV collagens, are often found in areas of high mechanical stress, and have roles in fibrillogenesis and maintaining the integrity and mechanical properties of the tissue [115].

Type XVI and XXII collagens belong to the group of FACITs collagens. Type XVI collagen has been identified in the territorial matrix of the chondrocytes, associating with thin weakly banded collagen fibrils containing type II and XI collagen [116]. It may be incorporated into structurally and functionally discrete matrix aggregates in cartilage [13]. Type XXII collagen is expressed at the junction between synovial fluid and surface of articular cartilage and associated with the extrafibrillar matrix in cartilage [117].

Type XXVII collagen is a fibril-forming collagen. It is mainly localized at sites of transition from cartilage to bone, and in the matrix surrounding proliferative chondrocytes in the epiphyseal growth plate [118,119]. It is believed to play a key structural role in the ECM of the growth plate and is required for the organization of the proliferative zone [119].

### 4.3. Articular Collagen Types Synthetized in Pathological Processes

#### Type I Collagen

The collagen type I triple helix is formed as a heterotrimer by two α1(I) chains and one α2(I) chain [α1(I)2α2(I)], which are encoded by *COL1A1* and *COL1A2* genes [49,120].

Type I collagen is the most abundant collagen in the body. It forms more than 90% of the organic mass of bone and is the major collagen of tendons, skin, ligaments, cornea, and many interstitial connective tissues with the exception of very few tissues such as hyaline cartilage, brain and vitreous body [31,36]. It is synthesized in large quantities by fibroblasts, osteoblasts, and to a lesser extent by nearly all other tissue cells [36,121,122].

This collagen is always incorporated into heterofibrils containing either type III collagen in skin and reticular fibers, type V collagen in bone, tendon, cornea, and other tissues, or in heterofibrils containing both collagens [36,123,124]. Type I/III collagen heterofibrils are a constituent of reticular fibers of most parenchymal tissues such as lung, kidney, liver, muscle, or spleen, with the exception of hyaline cartilage, brain and vitreous humor [125].

In most organs and notably in tendons and fascia, type I collagen provides tensile stiffness and in bone, it defines considerable biomechanical properties concerning load bearing, tensile strength, and torsional stiffness, ensuring the stability and integrity of the tissues [31]. In addition to its biomechanical properties, type I collagen is important as adhesive substrate for many cells and plays a major role in organ and tissue development, in cell migration, proliferation and differentiation, and in wound healing, tissue remodeling and hemostasis [121].

Type I collagen is generally used as a marker for fibrous connective tissue, bone, and dentin [126,127]. Fibrocartilage is different from articular hyaline cartilage due to the presence of type I collagen and the lower content of glycosaminoglycans [128]. Pathological and disorganized regeneration of tissue in a variety of organs after injury often results in deposition of excessive fibrotic tissue with inferior biomechanical properties, as occurs with articular cartilage, where fibrocartilage is created as repair tissue [128,129]. Full-thickness disruption of articular cartilage by trauma to synovial joints is one example in which highly specialized hyaline cartilage is replaced by biomechanically inferior, disorganized fibrotic tissue enriched in collagen type I [130].

As mentioned above, hyaline articular cartilage contains type II collagen, fibrocartilage contains a mixture of type I and II collagens, and fibrous tissue contains type I collagen [127,128,129,131,132]. In this way, type II collagen cannot be used solely to determine whether a cartilage is hyaline type, since it can be also found in fibrocartilage, so it would be necessary to determine no type I collagen absence [133].

Mutations in the genes *COL1A1* and *COL1A2* cause of most cases of osteogenesis imperfecta. It is a heterogeneous hereditary disorder of bone matrix formation and remodeling that causes bone fragility and deformity, blue sclera, short structure, dentinogenesis imperfecta, and hearing loss [134,135].

### 4.4. Collagens in Pathological Situations

OA is a disease that is often associated with age, female gender, obesity, muscle weakness, and joint injuries [136]. This disease transforms the main collagen of the articular cartilage, type II collagen, into a mixture of type I, II and III collagens. It can induce a 100-fold upregulation of type I collagen and 6-fold of type III collagen, and a 5-fold downregulation of type II collagen [128]. Additionally, it was reported that a higher amount of type VI collagen is found in osteoarthritic cartilage that could be a consequence of a protective effect for chondrocytes [137]. Other authors also described an increase in type III and VI collagen in osteoarthritic articular cartilage, however they observed that a greater amount of type II collagen is also deposited [137,138].

The wound-healing role of major and minor collagens likely to play during the articular regeneration has not been completely deciphered, thus, more efforts should be made to elucidate this potential role.

## 5. Role of Collagen Fibrils in the Quality of Repaired Tissue in Chondral Defects

As mentioned above, the healthy articular cartilage is characterized by presenting a hyaline structure. Several authors have reported that using different current therapies for chondral lesions they have been able to obtain a hyaline-like repair tissue [139,140,141,142]. Thus, in this context, the basic question that is often asked regarding the cartilage regeneration after damage is about the role of all collagen types in the repair assessment, and the threshold to consider a regenerated cartilage as hyaline-like cartilage including ECM evaluation.

When evaluating the quality of a repaired cartilage, must be assessed the morphological and structural characteristics of the repaired regenerated tissue must be, and the presence and arrangement of collagens in the ECM.

### 5.1. Methods of Cartilage Tissue Reparation

There are several therapeutic modalities that aim to reduce pain and restoring cartilage function. These strategies can be medical treatments such as pharmacological therapy, or surgical treatments such as palliative therapies (e.g., chondroplasty and debridement), repair techniques (e.g., drilling and microfracture), restorative techniques (e.g., tissue engineering) or prosthetic replacement [90,142,143,144,145,146,147].

Debridement is used to reduce pain; however, there is no obvious physiological or pathological evidence to show that it is beneficial for cartilage repair [148]. Perforation and microfracture repair techniques are considered first-line treatments given their minimally invasive nature, technical ease, and low cost [142,143]. They can be used alone or in combination with other tissue engineering techniques [149]. Tissue engineering seeks to repair damaged cartilage by introducing an optimized combination of cells, scaffold, and bioactive factors that can be transplanted into a patient [150]. Biomaterial scaffolds or hydrogel are being made with collagens given their ability to promote cartilage formation, the most common being type I and II collagen hydrogels [151,152]. Some of the techniques most frequently used in tissue engineering to repair chondral defects are autologous chondrocyte implantation, osteochondral autograft transfer and osteochondral allograft [143,145].

Despite the large number of methods that exist to treat chondral defects, they cannot restore a normal cartilage, and one of the main limitations in fibrocartilage formation without lasting improvements [19,145]. Much information is unknown about the intrinsic repair processes of damaged cartilage, and consequently the search for a treatment that would restore normal articular cartilage. For this reason, it is so important to evaluate in depth the repair quality of the repaired cartilage, including an exhaustive assessment of collagens.

### 5.2. Methods for Evaluating Morphological and Structural Characteristics of a Repaired Cartilage

The assessment of the morphological and structural characteristics is carried out with histopathological techniques such us HE staining, toluidine blue and Safranin O/fast green staining.

There are several histopathological evaluation methods that help us determine how similar repaired cartilage is compared to healthy cartilage [131,133,153,154]. These are detail studies that allow us to obtain standardized assessment methods. In summary, all of them and most authors agree that therapies aimed at developing successful hyaline cartilage regeneration should obtain a smooth surface predominantly rounded, lagoon-organized chondrocytes of low cell density a repaired tissue capable of integrating with living native cartilage, and good condition of subchondral bone [11,15,17,24,25,155].

### 5.3. Methods Allowing Evaluating Collagen of a Repaired Cartilage

Collagens have been visualized using a variety of techniques, but the most common are histopathologic techniques because of they are less expensive and sample preparation is less complicated [131]. Toluidine blue and picrosirius red staining are the most widely used. It is important to observe these sections stained with polarized light to view the orientation of the collagen fibers [131,156,157]. In addition, it is very common to use immunohistochemical techniques that allow us to visualize the presence and location of a certain collagen in the cartilage. Immunohistochemically marked tissues are observed under an optical microscope [90,133]. Despite involving a more complicated preparation and being more expensive, some authors use transmission electron microscopy, atomic force microscopy or structural illuminating microscopy, obtaining a higher resolution visualization of collagen fibers [142,157,158].

Regarding assessment methods, there is no standardized evaluation that determine the intrinsic quality of all collagen types in the repaired cartilage. Some of the previously mentioned methods [131,133,153,154], make a brief reference to the main collagens (type II, IX, and XI collagens), but there is a lack of information in considering the rest of the collagens contained in the articular cartilage to evaluate the quality of the repair and whether this repaired tissue can be considered as hyaline cartilage.

Therefore, based on the information reported in several studies [3,12,13,14,24,50,51,56,74,75,76,79,82,90,95,102,106,107,108,113,114,115,116,117,118,119,127,128,130,131,132,133], the composition of main and minor collagen types in ECM to consider the quality of a repaired cartilage as hyaline-type cartilage should be:

- Main collagens:
  - 90–95% type II collagen distributed throughout all areas of the ECM.
  - 1–5% type IX collagen distributed throughout all areas of the ECM.
  - 1–5% type XI collagen distributed throughout all areas of the cartilage and around the chondrocytes.
- Minor collagens:
  - 1–2% type VI collagen distributed around the chondrocytes.
  - 1% type X collagen present only in the calcified area.
  - Presence of type IV and V collagen around the chondrocytes.
  - Possible presence of type III, XII, XIV, XVI, XXII, and XXVII collagens.
- Absence of type I collagen.

## 6. Conclusions

There are many types of collagens present in the articular cartilage, both main and minor collagens, all of which are crucial for maintaining cartilage health. To accurately determine the repair tissue quality after a chondral lesion, the complete structure of the repaired cartilage tissue, including cells and their environment and main and minor collagen types in ECM, should be included in the histopathological evaluation methods.

## Figures and Tables

**Table 1:** Types of collagens that may be present in articular cartilage.

Collagen	Chains	Genes	Clasification	% *	Distribution in Articular Cartilage	Distribution in the Human Body
Type I	[α1(I)]_2_α2(I)	*COL1A1 COL1A2*	Fibrill-forming collagen	0%	Fibrocartilage	Bone, skin, cornea, and many interstitial connective tissues with the exception of hyaline cartilage, brain and vitreous body.
Type II	[α1(II)]_3_	*COL2A1*	Fibrill-forming collagen	90–95%	ECM of all zones	Cartilage, vitreous, and intervertebral disc.
Type III	[α1(III)]_3_	*COL3A1*	Fibrill-forming collagen	n/a	n/a	Bloods vessels, uterus, bowel, skin, tendon, ligament, cartilage, periodontal ligament, and synovial membranes.
Type IV	[α1(IV)]_2_α2(IV) α3(IV)α4(IV)α5(IV) [α5(IV)]_2_α6(IV)	*COL4A1 COL4A2 COL4A3 COL4A4 COL4A5 COL4A6*	Network-forming collagen	n/a	PCM	Skin, basement membranes, lung, kidney, cochlea eye, smooth muscle, oesophagus, and cartilage.
Type V	α1(V)_2_α2(V)	*COL5A1 COL5A2*	Fibrill-forming collagen	n/a	PCM	Adipose tissue, skeletal muscle, cartilage, pancreatic islets, skin, placenta, and lung.
Type VI	α1(VI)α2(V)α3(V) α1(VI)α2(V)α4(V) α1(VI)α2(V)α5(V) α1(VI)α2(V)α6(V)	*COL6A1 COL6A2 COL6A3 COL6A4 COL6A5 COL6A6*	Beaded filament collagen	1–2%	PCM	Skin, cornea, blood vessels, heart, lung, adipose tissue, nervous, pancreas, bone, cartilage, and muscle.
Type IX	α1(IX)α2(IX)α3(IX)	*COL9A1 COL9A2 COL9A3*	FACIT	1–5%	ECM of all zones and growth plate in adults	Cartilages, eye vitreum, avian cornea, ear, and intervertebral disc.
Type X	[α1(X)]_3_	*COL10A1*	Network-forming collagen	1%	Calcified zone and hypertrophic cartilage	Hypertrophic cartilage and the calcified zone.
Type XI	α1(XI)α2(XI)α3(XI)	*COL11A1 COL11A2 COL2A1*	Fibrill-forming collagen	1–5%	ECM of all zones and PCM	Cartilage, tendons, trabecular bone, testis, trachea, skeletal muscle, placenta, ovarian, lung, and brain.

*: percentage of collagen in healthy articular cartilage; ECM: extracellular matrix; PCM: pericellular matrix; FACIT: fibril-associated collagens with interrupted triple helices. n/a: no studies have been found to support it.

## Abbreviations

ECM	Extracellular matrix
FACITs	Fibril-associated collagens with interrupted tripled helices
MACITs	membrane-associated collagens with interrupted tripled helices
MMP13	Matrix metalloproteinase 13
OA	Osteoarthritis
PCM	Pericellular matrix
PG	Proteoglycan
RER	Rough endoplasmic reticulum
SMD	Spondylometaphyseal dysplasia
